# Risk of neuropsychiatric adverse events associated with varenicline treatment for smoking cessation among Dutch population: A sequence symmetry analysis

**DOI:** 10.1002/pds.5351

**Published:** 2021-09-09

**Authors:** Yuanyuan Wang, Job F. M. van Boven, Jens H. J. Bos, Catharina C. M. Schuiling‐Veninga, H. Marike Boezen, Bob Wilffert, Eelko Hak

**Affiliations:** ^1^ Department of PharmacoTherapy, ‐Epidemiology and ‐Economics Groningen Research Institute of Pharmacy, University of Groningen Groningen The Netherlands; ^2^ Groningen Research Institute for Asthma and COPD (GRIAC) University Medical Center Groningen, University of Groningen Groningen Netherlands; ^3^ Department of Clinical Pharmacy and Pharmacology University Medical Center Groningen, University of Groningen Groningen The Netherlands; ^4^ Department of Epidemiology University Medical Center Groningen, University of Groningen Groningen The Netherlands

**Keywords:** anxiety, depression, neuropsychiatric adverse events, sequence symmetry analysis, sleep disorders, varenicline

## Abstract

**Purpose:**

Varenicline is an effective treatment for smoking cessation. While clinical trials did not confirm a causal role, case reports suggested a possible link of varenicline with neuropsychiatric adverse drug events (NPAEs). This study aims to investigate the risk of NPAEs associated with varenicline initiation among the general population in a real‐world setting.

**Methods:**

We conducted a sequence symmetry analysis (SSA) based on the University of Groningen IADB.nl prescription database. We selected incident users of both varenicline and marker drugs for NPAEs, including depression, anxiety and sleep disorder within different time‐intervals. Adjusted sequence ratios (aSR) were calculated for each time‐interval.

**Results:**

Within 365‐days' time‐interval 1066 patients were incident users of both varenicline and NPAE marker drugs. In total, 505 patients were prescribed varenicline before NPAE marker drugs and 561 vice versa (crude sequence ratio [cSR] 0.90, 95% CI: 0.80–1.02). After adjustments for trends in prescriptions, overall a null association was found (aSR 1.00, 95% CI: 0.89–1.13). Regarding specific NPAEs, no increased risks were found for depression nor anxiety within any time‐interval. A small transient increased risk was found for sleep disorders, particularly in earlier time‐intervals 3 and 6 months (aSRs 1.52, 95% CI: 1.10–2.11 and 1.45, 95% CI: 1.15–1.83, respectively). Subgroup and sensitivity analyses showed similar findings.

**Conclusions:**

Varenicline initiation was unlikely to be associated with an increased risk of taking anti‐depressants nor anti‐anxiety drugs. Yet a small, but statistically significant, transient association with drugs for sleep disorders was noticed, possibly associated with withdrawal symptoms caused by smoking cessation.

**Key Points**

- In a real‐world setting, initiation of varenicline was not associated with an increased risk of severe adverse events like depressions or anxieties.
- A possible transient increase in sleep disorders should be taken into account to minimize its influence on the drug adherence.
- The evidence on neuropsychiatric safety of varenicline from this observational self‐controlled sequence symmetry analysis (SSA) design is consistent with evidence from randomized controlled trials.

## 1 INTRODUCTION

Although the prevalence of tobacco use has been declining in recent years,^1^ the tobacco epidemic is still one of the largest public health threats, related to more than 8 million deaths worldwide each year.^2^ Smoking‐related health problems are associated with a high burden for both family and society.^3^ To help halt this burden, varenicline as a first‐line pharmacological smoking cessation treatment (PSCT) was approved by the US Food and Drug Administration (FDA) in 2006. It has a unique mechanism of action compared with other PSCTs by acting as a partial agonist/antagonist with affinity and selectivity for α_4_β_2_ nicotinic acetylcholine receptors.^4^ In several randomized clinical trials (RCTs), varenicline was more effective for smoking cessation than bupropion, nicotine replacement therapy (NRT) or placebo.^5^, ^6^, ^7^

However, subsequent post‐marketing reports related to neuropsychiatric adverse events (NPAEs), such as depression, anxiety, sleep disorder and even suicide, among varenicline users raised concerns about the neuropsychiatric safety of varenicline.^8^ Based on the post‐marketing surveillance reports, the FDA placed a black box warning on varenicline about its risk of NPAEs in 2009.^9^ The individual case safety reports (ICSRs) and the FDA warning may have changed the attitude in both smokers and physicians towards varenicline even though the causal role of varenicline in inducing NPAEs could not be established then.^10^ Such information undoubtedly has led to the underutilization of varenicline for smoking cessation.^11^, ^12^, ^13^

Although ICSRs are important for signal detection of adverse drug reactions (ADRs), it is difficult to establish the association and compute incidence rates or risks.^14^, ^15^ Therefore, to identify the causal association between varenicline and risk of NPAEs, several cohort studies,^16^, ^17^, ^18^, ^19^, ^20^ as well as RCTs,^7^, ^21^, ^22^ were conducted after the safety warning. Notably, the synthesized evidence did not confirm the reports from ICSRs about severe neuropsychiatric risk from varenicline use.^23^, ^24^, ^25^ Therefore, the warning about possible suicidal risk from varenicline was removed by FDA in 2016. In subsequent years, doubts remained regarding the decision to lift the FDA warning. In particular, considering the relatively healthy population and limited power to detect rare events in clinical trials, results from RCTs may not reflect the situation in the real‐world setting.

Therefore, assessing whether NPAEs occur by varenicline in the real‐world is necessary. To our knowledge, although several observational studies explored the real‐world neuropsychiatric safety,^17^, ^18^, ^19^, ^20^ their results are not so consistent with RCTs, one study found protective effects of varenicline towards depression,^17^ the other reported increased risk towards anxiety and mood disorder in high risk population.^18^ Besides, these observational studies could not exterminate the influence of time‐invariant risk factors.^26^ Of note, sequence symmetry analysis (SSA), a self‐controlled study design, could offer the Supplementary real‐world evidence. Different from conventional observational studies, SSA can not only control genetic and other time‐invariant confounding effectively, but could also offer the relatively pure risk of NAPEs by varenicline and the timing of these NPAEs by varenicline if related association exists due to its unique design.

However, the relationship between varenicline and NPAEs has been studied before neither by SSA design, nor among the Dutch population. SSA has been widely used in recent decade to investigate the ADRs of medications as a signal detection method for pharmacovigilance,^27^ with moderate sensitivity, high specificity and robust performance.^28^, ^29^, ^30^ Unlike ICSRs, SSA uses electronic data to assess the association between a medication and an ADR by examining the symmetry in the sequence of index medication and marker medications as proxy for ADRs within a specific time window.^29^, ^31^

This study was to examine whether there is an association between varenicline use and onset of NPAEs in a real‐world setting among Dutch population by using SSA.

## 2 METHODS

### 2.1 Data source and setting

This study was conducted using the widely researched University of Groningen's pharmacy prescription database IADB.nl, a growing database that comprises a population of approximately 730 000 people from 72 community pharmacies in the northern Netherlands since 1994.^32^ The individuals are representative of the Dutch population with respect to drug use. Detailed prescription information includes date of prescription, name of dispensed drug, dosage, duration and related Anatomical Therapeutic Chemical (ATC) code of prescribed drug, but also year of birth and gender. As Dutch patients usually register at one single community pharmacy, the prescription information from pharmacies are relatively complete. Of note, over‐the‐counter drugs and prescriptions during hospital stay are not included in IADB.nl. Data after registration of varenicline from 2007 to 2018 were used for this study. The IADB.nl has been used in several previous SSAs.^33^, ^34^

### 2.2 Study design

The SSA design compares the frequency of initiation of a marker drug (as proxy for an ADR) before and after initiation of an index drug within the same individual. The individuals who were prescribed the index drug before marker drugs were labeled the “exposure → outcome” group. Conversely, those who were prescribed index drug after the marker drugs were labeled the “outcome → exposure” group. The crude sequence ratio (cSR) was the number of patients in the “exposure → outcome” group divided by the number of patients in the “outcome → exposure” group. If there is no association, the distribution of sequence orders is expected to be symmetrical and the cSR is close to 1. Of note, the SSA design is sensitive to changing trends in drug prescriptions over time due to factors like reimbursement policy changes and safety warnings. Therefore, a null‐effect sequence ratio (nSR) was used to adjust for the temporal prescription trends of index and marker drugs for ADRs.^31^ The adjusted sequence ratio (aSR) was calculated by dividing the cSR by the nSR.

### 2.3 Run‐in period

As the goal of SSA is to evaluate the relation between two incident events, we needed to identify the incident users of both index and marker drugs. As our index drug, that is, varenicline, was authorized for use by EMA on 26 September 2006, no varenicline was prescribed in the database IADB.nl before 2007. Therefore, in theory, patients with the first recorded prescription of varenicline during the study period were all incident users. Since the marker drugs have long been used for chronic treatment of NPAEs, most of their current users will be captured at the beginning of the study period. To exclude the current users and identify incident users of NPAEs marker drugs, we used the waiting time distribution to determine the run‐in period.^35^ Three months was used as run‐in period in this study as the waiting time distribution curves showed a steeply descending limb within first 3 months.

### 2.4 Study population and time interval

This study included all individuals who were incident users of both varenicline (ATC: N07BA03) and any marker drugs as potential treatment for NPAEs including depression (N06B, N06CA), anxiety (N05B) and sleep disorder (N05C) from January 2007 to December 2018 in IADB.nl. The first prescription of varenicline was the index date of varenicline, the first prescription of any of the marker drugs for NPAEs was set as index date of any NPAEs. The first prescription of specific marker drugs for depression, anxiety and sleep disorder were set as index date of specific NPAE. Those who were prescribed the index and marker drug on the same day were excluded as their sequence of varenicline and NPAE is not clear.

We defined different time‐intervals (365, 180, 90, 60, and 30 days) between the initiation of the varenicline and NPAEs marker drugs to explore their association. Therefore, incident users of both varenicline and marker drugs within pre‐set time intervals of each other were included for the SSA.

### 2.5 Statistical analysis

In this study, cSR was ratio of the number of patients in the “varenicline → NPAE” group to the number of individuals in the “NPAEs → varenicline” group. nSR is the expected SR in the absence of an associations due to the trends in utilization alone.^30^ Therefore, the aSR is calculated as the ratio of cSR to null‐effect SR (cSR/nSR). The detailed formula was as follows:

(1) cSR=number of patients in the“varenicline→NPAE”group/number of patients in the“NPAEs→varenicline”group.

(2) $$\mathrm{nSR}=P_{\alpha }/1-P_{\alpha },P_{\alpha }=\frac{\sum_{m\;=\;1}^{u}\left[A_{m}\left(\sum_{n\;=\;m\;+\;1}^{m\;+\;d}B_{n}\right)\right]}{\sum_{m\;=\;1}^{u}\left[A_{m}\left(\sum_{n\;=\;m-d}^{m-1}B_{n}\;+\;\sum_{n\;=\;m\;+\;1}^{m\;+\;d}B_{n}\right)\right]}$$

In the above formula, *u* is the last day of the research period, *m* and *n* are the consecutive days of the survey period, *d* is the time interval between index and marker drugs. *A*_m_ is the number of individuals being prescribed the index drug first on the *m* day. *B*_n_ is the number of individuals being prescribed marker drugs first on the *n* day.

(3) aSR=cSR/nSR

Confidence intervals (95% CI) of cSR and aSR were calculated by using the binomial distribution as follows:

$$95\%\mathrm{CI}=e^{\text{In}\left(\mathrm{SR}\right)\pm 1.96\mathrm{SE}}$$

where,

$$\mathrm{SE}=\sqrt{\frac{1}{\text{number of varenicline}\to \text{NPAE group}}+\frac{1}{\text{number of NPAEs}\to \text{varenicline group}.}}$$

All statistical analyses were performed using IBM SPSS statistics 25 (IBM Corporation, Armonk, NY) for Windows. We defined *p* < 0.05 as the level of statistical significance. All statistical tests were two‐tailed.

### 2.6 Subgroup and Sensitivity analyses

Stratified analysis was conducted according to different gender and age groups. We considered that several policy changes occurred during the study period: (a) reimbursement of PSCTs: PSCTs were reimbursed in 2011, non‐reimbursed in 2012 and again reimbursed from 2013 onwards. (b) Black box warning: FDA communicated it in 2009 and removed it in 2016; we performed sensitivity analyses by calculating the aSR for each year of the study period, as well as in several year groups. Besides, these sensitivity analyses stratified by specific year also reflected the influence of longer run‐in period on results as the incident users that captured before that year were all excluded. Thus, these analyses could further test the robustness of the results.

## 3 RESULTS

**FIGURE 1:**
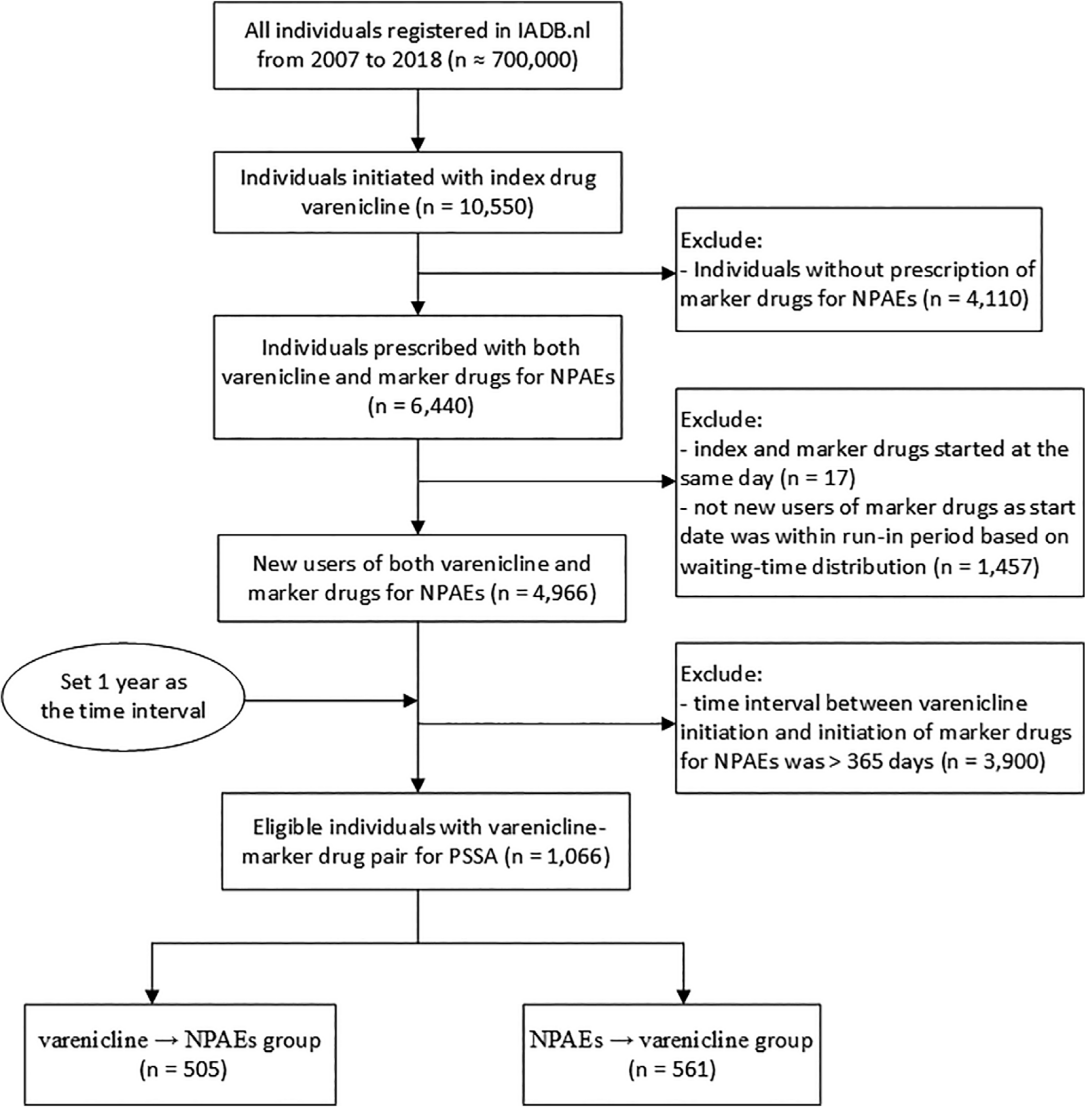
Flow chart of study population selection

**FIGURE 2:**
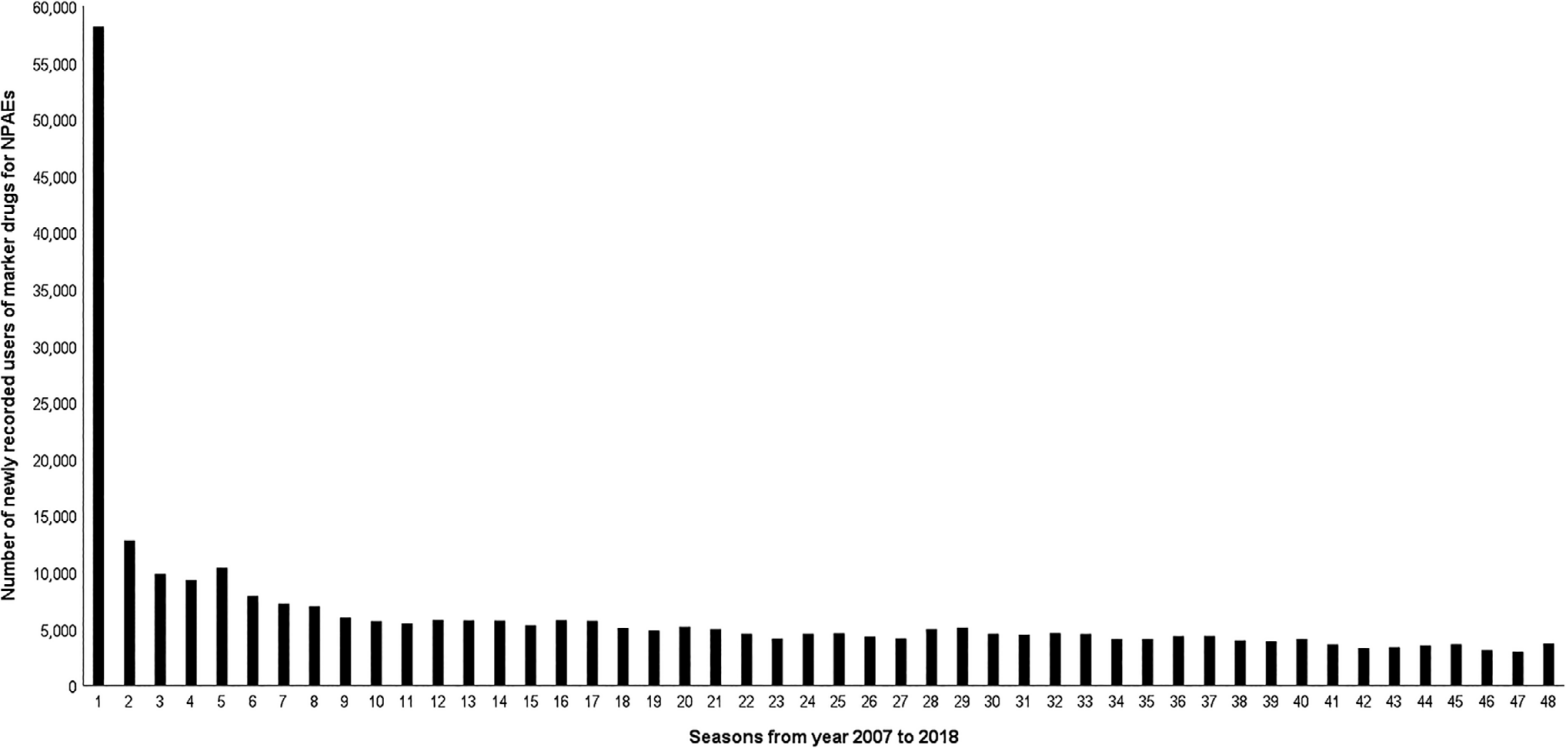
Waiting time distribution of the first prescriptions of marker drugs for NPAEs with the first year of study period

In total, there were 6440 patients who initiated both varenicline and marker drugs for the selected NPAEs (Figure 1). Of these, 17 patients were excluded because they were prescribed varenicline and marker drugs at the same day and 1457 patients were excluded as they were captured within the run‐in period of the first 3 months and may not be new users of marker drugs as shown in the waiting‐time distribution (Figure 2) that there was a steep decrease at the beginning of the study period after which a more or less stable situation was reached after 3 months, that is, when prevalent users were basically not in the newly captured population. Finally, for our SSA there were 1066 patients who were incident users of both varenicline and marker drugs that were prescribed within a 1‐year time period of each other.

Among the eligible patients, there were 505 patients in the varenicline → NPAE group and 561 patients in the NPAE → varenicline group. Within time‐interval of 365 days, no statistically significant difference was observed between the two groups. The aSR between varenicline and any NPAEs was 1.00 (95% CI: 0.89–1.13, Table 1). Also, no statistical significant association was observed between varenicline and depression (aSR 1.09, 95% CI: 0.94–1.26) nor anxiety (aSR 0.98, 95%CI: 0.85–1.14). There was, however, a small statistically significant increased risk of sleep disorders observed associated with varenicline (aSR 1.25, 95% CI: 1.05–1.48).

**TABLE 1:** Results of the sequence symmetry analysis for the association between varenicline use and marker drugs use for NPAEs by different time periods

NPAEs	Population (n)	Varenicline‐to‐NPAE group (n)	NPAE‐to‐varenicline group (n)	cSR (95% CI)	nSR	aSR (95% CI)
Within 365 days						
Overall	1066	505	561	0.90 [0.80, 1.02]	0.900	1.00 [0.89, 1.13]
Depression	727	364	363	1.00 [0.87, 1.16]	0.924	1.09 [0.94, 1.26]
Anxiety	716	335	381	0.88 [0.76, 1.02]	0.893	0.98 [0.85, 1.14]
Sleep disorder	532	286	246	1.16 [0.98, 1.38]	0.931	1.25 [1.05, 1.48]
Within 180 days						
Overall	603	322	281	1.15 [0.98, 1.34]	0.949	1.21 [1.03, 1.42]
Depression	389	208	181	1.15 [0.94, 1.40]	0.964	1.19 [0.98, 1.45]
Anxiety	394	206	188	1.10 [0.90, 1.34]	0.943	1.16 [0.95, 1.42]
Sleep disorder	295	172	123	1.40 [1.11, 1.76]	0.965	1.45 [1.15, 1.83]
Within 90 days						
Overall	315	173	142	1.22 [0.98,1.52]	0.975	1.25 [1.00, 1.56]
Depression	209	110	99	1.11 [0.85, 1.46]	0.984	1.13 [0.86, 1.48]
Anxiety	189	99	90	1.10 [0.83, 1.46]	0.971	1.13 [0.85, 1.51]
Sleep disorder	150	90	60	1.50 [1.08, 2.08]	0.984	1.52 [1.10, 2.11]
Within 60 days						
Overall	212	110	102	1.08 [0.82, 1.41]	0.982	1.10 [0.84, 1.44]
Depression	138	62	76	0.82 [0.58, 1.14]	0.989	0.83 [0.59, 1.15]
Anxiety	137	72	65	1.11 [0.79, 1.55]	0.979	1.13 [0.81, 1.58]
Sleep disorder	101	56	45	1.24 [0.84, 1.84]	0.990	1.26 [0.85, 1.86]
Within 30 days						
Overall	111	54	57	0.95 [0.65,1.37]	0.988	0.96 [0.66, 1.39]
Depression	74	31	43	0.72 [0.45, 1.14]	0.993	0.73 [0.46, 1.15]
Anxiety	73	32	41	0.76 [0.47, 1.21]	0.986	0.77 [0.48, 1.22]
Sleep disorder	50	25	25	1.00 [0.57, 1.74]	0.993	1.01 [0.58, 1.77]

Abbreviations: aSR, adjusted sequence ratio; CI, confidence interval; cSR, crude sequence ratio; NPAEs, neuropsychiatric adverse events; nSR, null‐effect sequence ratio.

**FIGURE 3:**
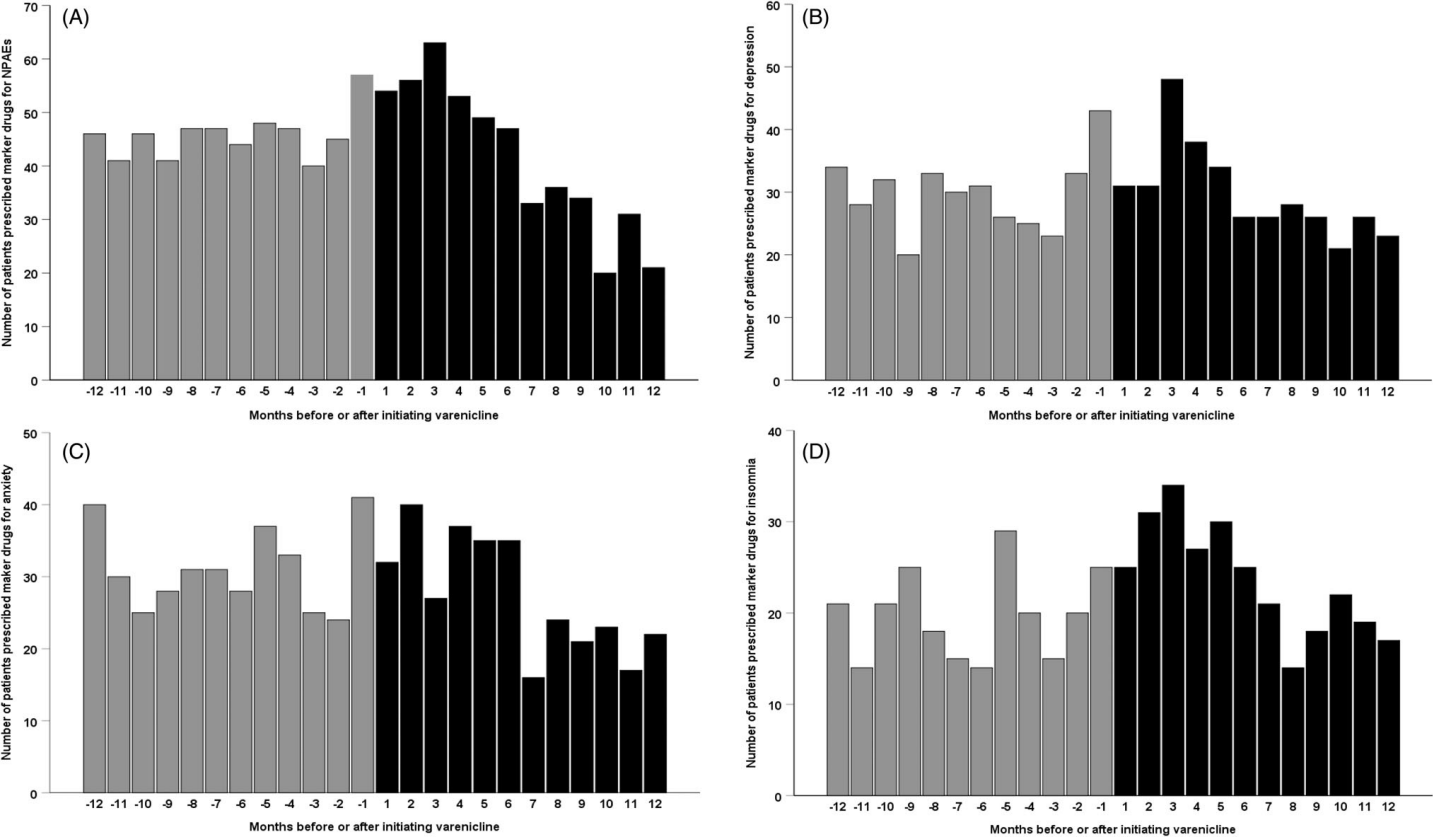
Frequency distribution of patients with (A) all NPAEs; (B) depression; (C) anxiety; (D) sleep disorder by number of months before or after the initiation of varenicline within 1 year

When we considered different time‐intervals between initiation of varenicline and any NPAE marker drugs, we also did not find significant associations (Table 1) within 30 days (aSR 0.96, 95% CI: 0.66–1.39) and 60 days (aSR 1.10, 95% CI: 0.84–1.44). There was a boundary significant increased risk of NPAEs observed with varenicline within 90 and 180 days. For the specific NPAEs, similar to the results observed within 365 days, no significant associations were observed between varenicline and specific NPAEs in depression and anxiety. Again, sleep disorder was the exception with an aSR of 1.52 (95% CI: 1.10–2.11) and 1.45 (95% CI: 1.15–1.83) within 90 and 180 days, respectively. Frequency distributions of patients with any or specific NPAE are shown in Figure 3.

In stratified analyses by gender and age groups (Table S1), a significant association between varenicline and sleep disorder was only seen in female and older age groups. Of note, a boundary significant small risk of depression associated with varenicline was also observed in older age groups (aSR 1.23, 95% CI: 1.01–1.50). As shown in Figure S1, there were three main fluctuations in the curve for the number of patients newly prescribed varenicline by years of study period. There was a high rise in newly prescribed varenicline in 2011 and a drop in 2012, followed by a sharp increase in 2013. Also, there was a small increase in newly prescribed varenicline from 2015 to 2016. In the results of sensitivity analysis by year groups or each year for the aSR between varenicline and overall NPAEs, we did not observe any statistically significant difference for the order of varenicline → NPAE and NPAE → varenicline groups, except for year 2011 (aSR 1.72, 95% CI: 1.27–2.32, Table S2).

## 4 DISCUSSION

This is the first study to assess the risk of NPAEs associated with varenicline using a SSA design. Based on the results of this study, we further confirm that no statistically significant increased risk of anxiety and depression treated with medications was associated with varenicline in all different time‐intervals. However, within 3, 6, and 12 months, there was a small, but statistically significant, increased risk of sleep disorder. We did not observe significant risk of sleep disorder associated with varenicline within shorter time‐intervals.

Our results are consistent with a meta‐analysis of RCTs published in 2015,^23^ regarding mood change and sleeping disorders. In this meta‐analysis compared with placebo (smokers, non‐smokers), there was no evidence of an increased risk of depression and even a reduced risk of anxiety among varenicline. Oppositely, a higher risk of sleep problems (e.g., insomnia, abnormal dreams) was observed in this meta‐analysis. In two large cohort studies, there was also no increased depression risk observed among varenicline users.^16^, ^17^ However, in a third cohort study, varenicline was found to be associated with a small increase in the risk of anxiety and mood conditions, although this was only observed in people with previous psychiatric disorders.^18^ Of note, different with RCTs with placebo, most cohort studies used NRTs as the reference to explore the risk of NPAEs associated with varenicline. NRTs may control the withdrawal symptoms better than placebo, but it could be vulnerable to some bias if the users' characteristics between varenicline and NRTs were different.

Sleep disorder is a commonly reported ADR associated with varenicline with an incidence ranging from 14.0% to 37.2%.^4^ It was also the most frequently reported NPAEs according to a prescription‐event monitoring study in England.^8^ Indeed, difficulty falling asleep and increased number of awakenings are common symptoms of nicotine withdrawal.^36^ In this study, we observed increased risk of sleeping disorders by varenicline, which could not be untangled from nicotine withdrawal effect as those in varenicline → NPAEs group would have more pronounced withdrawal effect compared with those in NPAEs → varenicline group. Although sleep disorder is not a serious ADR, it may result in poor adherence to varenicline and therefore potentially affect the possibility of quitting successfully. As such, clinicians should pay particular attention to this kind of side effect among varenicline users.

Of note, there was a traditional self‐controlled analysis conducted by Gershon et al. in 2018,^37^ that compared the relative incidence of more severe NPAEs in hospitalizations and emergency department visits during the period of varenicline use compared to the period without varenicline use. The relative incidence (RI) of NPAEs was slightly increased with a boundary statistical significance (RI 1.06; 95%CI: 1.00–1.13). Different from this study that focused on severe NPAE among inpatients, we focused more on mild NPAEs that happened among outpatients by using a different self‐controlled study design (i.e., SSA). Combining the results from our study and the study by Gershon et al, provides complimentary varenicline safety evidence for the general population among different real‐world settings.

Considering SSA is sensitive to prescribing trend over time,^31^ we did sensitivity analyses by calculating the aSR in each year of the study period. We observed fairly consistent results with our overall finding except for the year 2011 with a slightly increased risk of NPAEs, which may be influenced by PSCT reimbursement policy changes in the Netherlands since PSCTs were reimbursed in 2011 and the reimbursement was temporarily discontinued in 2012,^38^ thus more patients will fall into the “varenicline →NPAEs” group. At the same time, the FDA warning since 2009 may also gradually increase the concern about possible risk of NPAE that led by varenicline, which will lead to the prescription of varenicline was withheld for high‐risk patients. Thus, less patients will fall into the “NPAEs → varenicline” group and this will also result in the overestimate the SR, which will trend to be higher than 1. These above‐mentioned policies together will have an overall influence on the result estimation in the direction of an overestimation. Although nSR could adjust for changes in prescribing trends over time, it could not totally eliminate the influence of market trends of medicine use, especially for the sharp change. Of note, as we used 3 months as run‐in period to capture the incident users of marker drugs, the misclassification of prevalent users for incident users may not be totally excluded. However, its influence on total results should be very small considering the small number of eligible patients that captured in 2007 and the aSR in that year is rather consistent with those from the following 2 years. It's reported that females and older people are more sensitive to NPAEs.^39^, ^40^, ^41^ However, we did not find a significant risk of any NPAEs associated with varenicline in these sub‐groups except for sleep disorders, which is consistent with our original outcomes and showed the robustness of our results.

Our study has several strengths. SSA inherently controls for time‐invariant, patient‐specific confounders (e.g., sociodemographic, genetic and lifestyle‐related factors) compared with traditional observational studies.^28^ Second, this is the first study to assess the safety of varenicline by using SSA among Dutch population based on a large prescription database in the Netherlands, which is representative for a general, unselected, population. Third, due to our design we went beyond the question whether varenicline‐related ADRs occurred, and could also provide in‐depth analysis of their timing. Besides, compared with cohort studies using NRTs as comparator, SSA as a self‐controlled study could reflect the pure risk of varenicline by using “non‐use” as a comparator. Our study also has several potential limitations. First, SSA is sensitive to time‐varying confounding like disease severity, to minimize this time‐varying bias, we limited the time window between index and marker drugs to a maximum of 12 months and did sensitivity analyses by limiting time interval to even 3 and 6 months. Furthermore, due to absence of diagnostic data, marker drugs were used as proxy for NPAEs, there are some NPAEs (i.e., behavioral symptoms) that may not be treated by these maker drugs. Lastly, not all reported ADRs, like cardiovascular events or seizures,^17^, ^42^ were evaluated in this study. Some severe NPAEs like suicide, neuropsychiatric hospitalizations and emergency department visits could not be evaluated due to the data limitation. Despite these limitations are inherent to SSA methods and common in real‐world data sources, this study provides good supplementary evidence for the risk of NPAEs associated with varenicline use in a real‐word setting.

Our results by using SSA further confirmed the neuropsychiatric safety of varenicline about possible risk of depression and anxiety. Clinicians and users of varenicline should however remain aware of increased occurrence of sleep disorder, especially in the first 3–6 months after varenicline initiation, which may be the withdrawal symptoms. However, as sleep disorders could be early symptoms of depression,^43^, ^44^ it may also further result in increased smoking cessation treatment uptake, adherence and, ultimately, cessation rates. Proper education on expected timing of this event and personalized coping strategies is needed.

## 5 CONCLUSIONS

Our SSA results suggest that the real‐world use of varenicline is unlikely to be associated with risk of depression or anxiety that treated with medication. However, consistent with previous evidence, there was a small transient increased risk of sleep disorder associated with varenicline initiation, particularly in the first 3–6 months. Whether sleep disorder was caused by the adverse effects of varenicline or related to withdrawal symptoms needs confirmation by further studies.

## CONFLICT OF INTEREST

The authors declare no conflicts of interest.

## ETHICS STATEMENT

The authors state that no ethical approval was needed as this study was based on existing database, which was established before according to related ethics criteria.

## Supporting information


**Figure S1** Number of patients newly prescribed varenicline in each year of the study period.
**Table S1**: Prescription sequence symmetry results of the association between varenilcine use and marker drugs for NPAEs within a time window of 1 year, stratified by year.
**Table S2**: Prescription sequence symmetry results of the association between varenicline use and marker drugs for NPAEs within a time window of 365 days, stratified by gender and age groups.
