# Supplementary material for: Risk of neuropsychiatric adverse events associated with varenicline treatment for smoking cessation among Dutch population: A sequence symmetry analysis
Source: Pharmacoepidemiol Drug Saf. 2021 Sep 9;31(2):158–66. doi: 10.1002/pds.5351 (PMC9292305; doi:10.1002/pds.5351)
Supplement: Supplementary file 1 — Figure S1 Number of patients newly prescribed varenicline in each year of the study period. Table S1: Prescription sequence symmetry results of the association between varenilcine use and marker drugs for NPAEs within a time window of 1 year, stratified by year. Table S2: Prescription sequence symmetry results of the association between varenicline use and marker drugs for NPAEs within a time window of 365 days, stratified by gender and age groups. [file PDS-31-158-s001.pdf]

Supplementary materials

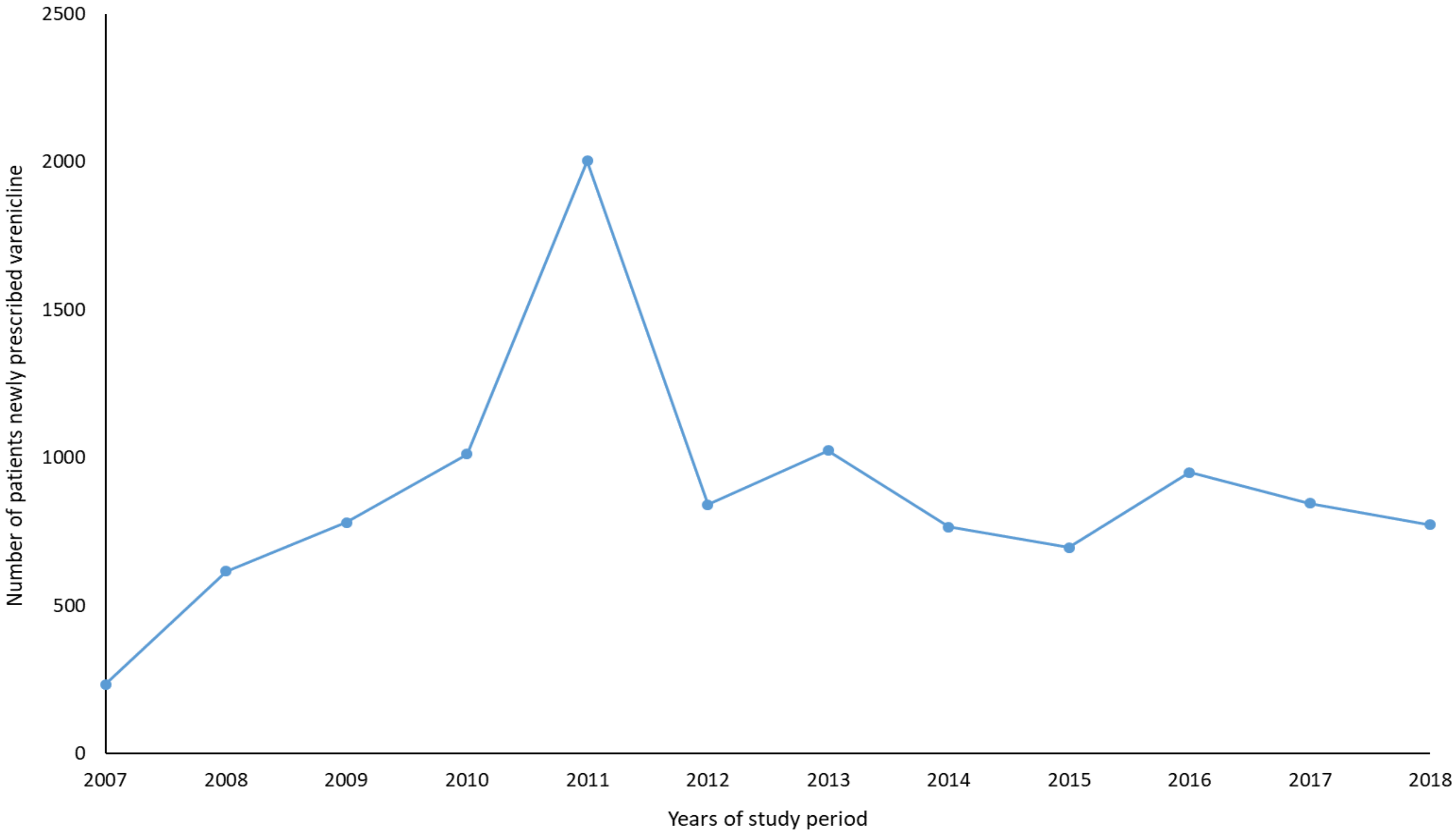

Figure S1. Number of patients newly prescribed varenicline in each year of the study period

Table S1. Prescription sequence symmetry results of the association between varenilcine use and marker drugs for NPAEs within a time window of 1 year, stratified by year.

Time periods	Number of patients*	Sequence order[a]	Crude SR	Null-effect SR	Adjusted SR
**Year groups**					
2007-2010	430	164/266	0.62 [0.51, 0.75]	0.75	0.82 [0.67, 1.00]
2011-2013	345	199/146	1.36 [1.10, 1.69]	1.10	1.23 [1.00, 1.53]
2014-2018	291	142/149	0.95 [0.76, 1.20]	0.96	0.99 [0.79, 1.25]
**Year**					
2007	75	19/56	0.34 [0.20, 0.57]	0.62	0.55 [0.33, 0.93]
2008	122	41/81	0.51 [0.35, 0.74]	0.70	0.72 [0.49, 1.05]
2009	103	42/61	0.69 [0.46, 1.02]	0.97	0.71 [0.48, 1.06]
2010	130	62/68	0.91 [0.65, 1.29]	0.98	0.93 [0.66, 1.32]
2011	173	99/74	1.34 [0.99, 1.81]	0.78	1.72 [1.27, 2.32]#
2012	73	44/29	1.52 [0.95, 2.42]	1.06	1.43 [0.89, 2.28]
2013	99	56/43	1.30 [0.88, 1.94]	1.27	1.02 [0.69, 1.52]
2014	67	30/37	0.81 [0.50, 1.31]	0.98	0.83 [0.51, 1.34]
2015	58	25/33	0.76 [0.45, 1.27]	0.92	0.83 [0.49, 1.39]
2016	65	36/29	1.24 [0.76, 2.02]	0.83	1.50 [0.92, 2.44]
2017	69	35/34	1.03 [0.64, 1.65]	1.08	0.95 [0.59, 1.52]
2018	32	16/16	1.00 [0.50, 2.00]	1.11	0.90 [0.45, 1.80]

*Patients with initial prescription of both index drug varenicline and marker drugs for NPAEs. #p<0.05, with statistical significance.

[a] the number of patients who initiated marker drugs for NPAEs after index drug varenicline divided by the number of patients who initiated varenicline after marker drugs for NPAEs. NPAEs: neuropsychiatric adverse events; SR: sequence ratio;

Table S2. Prescription sequence symmetry results of the association between varenicline use and marker drugs for NPAEs within a time window of 365 days, stratified by gender and age groups.

Variables	Number of patients*	Sequence order[a]	Crude SR	Null-effect SR	Adjusted SR
**Gender**					
Male					
Any NPAEs	529	254/275	0.92 [0.78, 1.10]	0.91	1.02 [0.86,1.21]
Depression	307	149/158	0.94 [0.75, 1.18]	0.92	1.02 [0.82, 1.28]
Anxiety	333	151/182	0.83 [0.67, 1.03]	0.90	0.92 [0.74, 1.15]
Sleep disorder	254	136/118	1.15 [0.90, 1.47]	0.94	1.22 [0.95, 1.56]
Female					
Any NPAEs	537	251/286	0.88 [0.74, 1.04]	0.90	0.98 [0.83, 1.16]
Depression	420	215/205	1.05 [0.87, 1.27]	0.93	1.13 [0.94, 1.37]
Anxiety	383	184/199	0.92 [0.76, 1.13]	0.89	1.04 [0.85, 1.27]
Sleep disorder	278	150/128	1.17 [0.93, 1.48]	0.92	1.27 [1.00, 1.61]#
**Age groups**					
< = 45 years					
Any NPAEs	476	214/262	0.82 [0.68, 0.98]	0.91	0.89 [0.75, 1.07]
Depression	326	151/175	0.86 [0.69, 1.07]	0.93	0.93 [0.75, 1.15]
Anxiety	323	136/187	0.73 [0.58, 0.91]	0.90	0.81 [0.65, 1.01]
Sleep disorder	234	116/118	0.98 [0.76, 1.27]	0.95	1.04 [0.80, 1.34]
> 45 years					
Any NPAEs	590	291/299	0.97 [0.83, 1.14]	0.89	1.10 [0.93, 1.29]
Depression	401	213/188	1.13 [0.93, 1.38]	0.92	1.23 [1.01, 1.50]
Anxiety	393	199/194	1.03 [0.84, 1.25]	0.89	1.16 [0.95, 1.41]
Sleep disorder	298	170/128	1.33 [1.06, 1.67]	0.92	1.44 [1.15, 1.82]#

*Patients with initial prescription of both index drug varenicline and maker drugs for NPAEs. [a] the number of patients who initiated marker drugs for NPAEs after index drug varenicline divided by the number of patients who initiated varenicline after marker drugs for NPAEs. #P<0.05; NPAEs: neuropsychiatric adverse events; SR: sequence ratio;
